# Effectiveness of Robot-Assisted Versus Conventional Occupational Therapy on Changes in Upper Extremity Function After Cervical Spinal Cord Injury (Armeo X-over Trial): Study Protocol of a Randomised Crossover Trial

**DOI:** 10.3390/mps9020031

**Published:** 2026-02-26

**Authors:** Chantal Wunderlin, Flavia Bürgisser, Armin Gemperli, Claudio Perret, Mario Widmer

**Affiliations:** 1 Neuro-Musculoskeletal Functioning and Mobility Group, Swiss Paraplegic Research, 6207 Nottwil, Switzerland; claudio.perret@paraplegie.ch (C.P.); mario.widmer@paraplegie.ch (M.W.); 2 Faculty of Health Sciences and Medicine, University of Lucerne, 6005 Lucerne, Switzerland; armin.gemperli@unilu.ch; 3 Department of Therapy, Swiss Paraplegic Centre, 6207 Nottwil, Switzerland; flavia.buergisser@paraplegie.ch

**Keywords:** spinal cord injury, quadriplegia, upper extremity, robotics, rehabilitation, occupational therapy

## Abstract

Robot-assisted therapy (RT) is increasingly implemented in rehabilitation, yet evidence on its effectiveness in improving upper extremity function after cervical spinal cord injury (cSCI) remains limited. Therefore, this randomised crossover study aims to investigate the effects of unilateral RT compared to conventional unilateral occupational therapy (OT) on upper extremity function in individuals with cSCI. 40 participants with traumatic or non-traumatic cSCI (16–81 days post-injury, neurological level of injury: C1–T1) will be randomised (1:1), stratified by their predicted recovery profile, to receive 6 weeks of RT (ArmeoSpring) and 6 weeks of OT in random order, each 3 × 30 min/week in addition to the clinical routine therapy. Assessments are conducted before (t_0_), between (t_1_) and after both intervention blocks (t_2_ and t_3_). The primary outcome is the Quantitative Grasping Subtest of the Graded Redefined Assessment of Strength, Sensibility, and Prehension (GRASSP-QtG); primary analysis uses a linear mixed model to estimate the treatment effect based on change scores. Recruitment is currently ongoing. This randomised crossover study allows the collection of a comprehensive dataset to generate knowledge about treatment effectiveness, enabling future individuals with cSCI to benefit from improved and individualised therapy schedules.

## 1. Introduction

Cervical spinal cord injury (cSCI) leads to motor and sensory impairments that limit upper extremity function. These impairments limit activities of daily living (ADLs) and reduce independence and quality of life. Individuals with cSCI consider improvements in upper extremity function to be one of the most significant factors for improving their quality of life [1]. Even small functional improvements can substantially increase independence in performing ADLs and community integration [1,2].

Improvements in function after cSCI may be induced by repetitive and activity-based training that induces practice-dependent brain and spinal plasticity [3]. To reach a high number of repetitions during therapy, specialised rehabilitation centres increasingly implement robot-assisted interventions into clinical practice, aiming to improve upper extremity function. Robotic devices provide individuals with cSCI with high-intensity, individualised therapy and motivating feedback (e.g., gamification), while saving personnel resources and closely monitoring therapy sessions. Robot-assisted therapy (RT) is safe and feasible [4], but has not yet proven clinically effective for individuals with cSCI [5].

The number of studies about RT for upper extremity rehabilitation has been on the increase since the early 2000s, yet clear evidence in individuals with cSCI remains limited. Most studies have focused on the stroke population, where RT yields comparable outcomes to those of conventional therapy [6]. In contrast, when narrowing the focus to RT for upper extremity rehabilitation after cSCI, the number of studies decreases; even fewer have followed a randomised-controlled trial (RCT) design.

One such study by Jung, et al. [7] combined the ArmeoPower (Hocoma AG, Volketswil, Switzerland) with the Amadeo (Tyromotion Inc., Graz, Austria). The study showed improvements in upper extremity motor function after five weeks of RT additional to a daily therapy routine. However, no differences emerged compared to additional occupational therapy (OT). Similarly, Kim, et al. [8] found no significant difference after four weeks of additional OT or RT using only the ArmeoPower. Moreover, a recent pilot RCT by Kilkki, et al. [9], employing the Amadeo, Diego and Pablo devices, reported no statistically significant motor improvements compared to no intervention. Importantly, all three RCTs included relatively small sample sizes (*n* = 34, *n* = 30 and *n* = 20) while using very broad inclusion criteria, including enrolment periods ranging from one to ten years post-injury. Such studies are further constrained when considering that most of the motor recovery depends on the initial American Spinal Injury Association Impairment Scale (AIS) [10] and occurs within the first 6–9 months.

Moreover, a study has shown that from Hocoma’s Armeo concept, the ArmeoSpring seems to best suit the needs of individuals with cSCI and their therapists [11]. However, initial research assessing its efficacy has yielded nuanced results. The pilot study of Zariffa, et al. [12] assessed the efficacy of ArmeoSpring therapy in individuals with cSCI, in which one upper extremity received the intervention and the contralateral extremity served as the control. Although this study did not find significant differences between the intervention and control upper extremities across the overall sample, a subgroup with residual hand function at baseline showed significantly greater improvement in the sensibility subtest of the Graded Redefined Assessment of Strength, Sensibility, and Prehension (GRASSP). Building on these findings, Lozano-Berrio, et al. [13] conducted a pilot RCT to further investigate the efficacy of RT on upper extremity function. While improvements were demonstrated in both the intervention and control groups, the intervention group receiving conventional therapy and ArmeoSpring experienced more clinically relevant changes than the control group receiving conventional therapy only.

Despite these promising first results, clear evidence of the effectiveness of robot-assisted interventions for individuals with cSCI remains limited. Due to heterogeneous study designs, small sample sizes, broad enrolment periods and different robotic devices, it is unclear whether RT and OT just have similar effects, or whether potential superior effects of RT have been blurred [14]. To address this gap, this randomised crossover study aims to systematically investigate the effects of RT compared to conventional OT on upper extremity function in individuals with intermediate cSCI. The objective is to determine the effect of 6 weeks of unilateral RT using the ArmeoSpring (Hocoma AG, Volketswil, Switzerland) on upper extremity function compared to 6 weeks of conventional unilateral OT, both provided as 3 × 30 min sessions per week in addition to the clinical routine therapy scheme. The primary outcome will be the Quantitative Grasping Subtest of the GRASSP (GRASSP-QtG). We hypothesise that 6 weeks of unilateral RT will lead to greater improvements in the GRASSP-QtG compared to 6 weeks of conventional unilateral OT.

## 2. Materials and Methods

### 2.1. Study Design

This randomised, two-period, crossover trial investigates the effects of RT in comparison to OT on upper extremity function in individuals with intermediate cSCI. The study protocol follows the Standard Protocol Items: Recommendations for Interventional Trials 2013 Statement [15] (Supplement S1). This publication is based on version 1.2 of the study protocol, dated 20 August 2025. Recruitment of participants has been ongoing since 10 June 2025, and data collection is estimated to end in June 2028. The study is conducted at the Swiss Paraplegic Centre (SPC) located in Nottwil, Switzerland.

**Figure 1:**
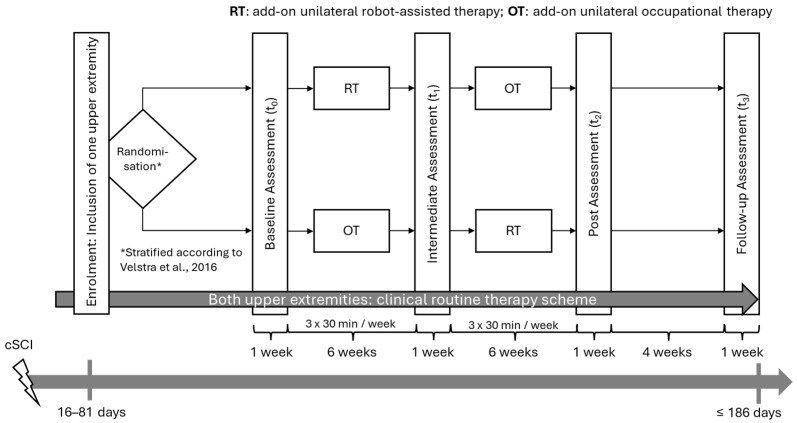
Randomised, two-period, crossover design. Participants are enrolled from day 16 to day 81 post-injury and undergo stratified randomisation by their predicted motor recovery [16] to one of the two intervention sequences (RT followed by OT or vice versa). One upper extremity is assigned to the intervention, but both upper extremities continue to receive clinical routine therapy. Each intervention block entails additional 3 × 30 min sessions per week for 6 weeks, with 1 week before (t_0_), in between (t_1_) and afterwards (t_2_) to assess the primary and secondary outcomes. t_3_ (≤day 186) will be conducted approximately 30 days after t_2_, but only if t_2_ is completed before day 150 after injury, in line with the European Multicentre Study about Spinal Cord Injury (EMSCI) study timeline [17]. The assessments at t_0_, t_1_, t_2_ and t_3_ last approximately 3 h each.

Participants are enrolled between day 16 and day 81 post-injury (Figure 1). The study takes 15 weeks, of which 12 weeks are devoted to therapy interventions and 3 weeks are devoted to assessments. In case the participants complete the intermediate assessment t_2_ before day 150 after injury, a follow-up assessment t_3_ of one week will take place around four weeks after t_2_, in line with the European Multicentre Study about Spinal Cord Injury (EMSCI) study timeline. With this, the study would take 20 weeks in total. The therapy interventions consist of RT as the experimental intervention and conventional OT as the control intervention. Both therapy interventions, RT and OT, are executed unilaterally and are additional to the clinical routine therapy scheme. Whereas the upper extremity included in the study is chosen by the participants together with their therapists, the sequence of the intervention blocks is randomly assigned. Each intervention block comprises 3 × 30 min training sessions per week for 6 weeks.

### 2.2. Participants and Randomisation

The study population comprises individuals with cSCI who have upper extremity impairments and are in the intermediate stage of recovery (2 weeks to 6 months post-injury). Eligible participants include adults (≥18 years) with traumatic or non-traumatic cSCI with a neurological level of injury (NLI) between C1 and T1 and classified as AIS grades A–D [18]. Detailed inclusion and exclusion criteria are provided in Table 1.

In total, 40 participants will be randomised to one of the two intervention sequences. Participants will be randomised 1:1 using a permuted block design with a block size of four, stratified by their predicted recovery profile [16]. It has been shown that upper extremity function at six months after onset of cSCI can be predicted using two specific upper extremity muscles. The two predictors are the Flexor Digitorum Profundus muscle of the International Standards for the Neurological Classification of Spinal Cord Injury (ISNCSCI-FDP) and the Musculus deltoideus pars acromialis of GRASSP (GRASSP-Delto) [16]. Both predictors will be assessed between day 16 and day 40 after onset of cSCI to derive the corresponding stratum for stratified randomisation. This will ensure well-balanced intervention sequences in terms of the predicted recovery potential and may facilitate the identification of participant subgroups that show particularly strong or weak responses to one of the interventions.

The stratified permuted block randomisation is implemented using the web-based data capture and management system secuTrial (iAS, Berlin, Germany; see Data management and safety provisions). The randomisation algorithm was configured by the data manager of the clinical trial unit (CTU) of Swiss Paraplegic Research, ensuring that the allocation sequence remains concealed from the investigators. Allocation is only revealed to qualified members of the study team after an enrolled participant’s randomisation is triggered within secuTrial. Therefore, concealment of allocation will be ensured.

### 2.3. Study Procedure

The CTU informs the study team when a patient who has been newly admitted to the SPC fulfils the main eligibility criteria. Patients who express interest in participating in the study receive verbal and written information (Supplement S2) about the study’s purpose, procedures, and potential risks from a qualified study team member. Potential participants are given at least 24 h to decide whether to participate. Written informed consent is obtained from all participants prior to enrolment.

The participants will be enrolled between day 16 and day 81 post-injury. Following enrolment, inclusion and exclusion criteria are verified, and the descriptive data (see Outcomes) of the participants are collected. Moreover, the stratification parameters ISNCSCI-FDP and GRASSP-Delto are extracted from the medical records and used for stratified randomisation. Furthermore, the participants together with their therapists decide which upper extremity to include in the study.

The baseline assessment t_0_ takes place within one week before the intervention begins and starts no later than day 82 after the injury. The intermediate t_1_ and post assessment t_2_ take place within one week after completion of the preceding intervention block. In case t_2_ is completed before day 150 after injury, the follow-up assessment t_3_ is conducted approximately 30 days after t_2_, but before day 186 after injury. Otherwise, the follow-up assessment t_3_ is omitted, and t_2_ has to be completed before day 186 after injury in line with the timepoints from the EMSCI study [17]. In every assessment, the primary and secondary outcomes (see Outcomes) are assessed by a team of assessors, all specifically trained in the study assessments. Each assessment lasts approximately three hours.

One week after starting the baseline assessment t_0_ (or t_1_ respectively), the randomly assigned intervention block starts (see Study interventions). The experimental intervention RT is conducted by members of the Armeo specialist group, which is specifically trained to deliver RT using Armeo devices at the SPC. The control intervention OT is conducted by experienced occupational therapists from the SPC. During both blocks, parameters characterising the interventions (see Further outcomes) and therapy adherence are documented by the therapists and study team.

For each completed assessment, the participants will receive a financial compensation of CHF 50. Hence, completion of all four assessments (t_0_–t_3_) will be compensated with CHF 200 per participant.

To ensure unbiased outcomes, the assessors are blinded to the intervention sequence. Members of the study team, therapists and participants are instructed not to disclose any information about the foregoing intervention blocks. In the event of unblinding, the assessors are instructed to inform the principal investigator about the unblinding. The assessor involved must not conduct any further assessments of the respective participant. Instead, another member of the assessment team will be assigned to perform the remaining assessments. Blinding of study participants and treating therapists is, however, not possible.

### 2.4. Study Interventions

Both interventions include three therapy sessions of 30 min per week for 6 weeks each. Depending on the schedule and availability of the patients and the therapists, the add-on therapy frequency can range between 2 and 4 sessions per week, not exceeding 18 therapy sessions in 6 weeks. Each session is scheduled for a 45 min time slot to ensure a therapy duration of 30 min. The first session is scheduled for 60 min to collaboratively define individualised and meaningful therapy goals regarding “body function”, “body structure” and the “activities” domain according to the International Classification of Functioning, Disability and Health (ICF). The goals are discussed and defined jointly by the participant, the participant’s lead therapist, and the therapist conducting the study intervention. In case of the experimental intervention RT, the 60 min in the first session are also used to adapt the device settings to each participant individually.

Participants continue to receive any concomitant care and medication they normally receive during rehabilitation. Only the experimental and control interventions as specifically described below are not permitted as concomitant therapy for the upper extremity included in the study. However, therapies of the other upper extremity are not restricted in this respect.

#### 2.4.1. Experimental Intervention RT

The experimental intervention consists of unilateral RT using the ArmeoSpring, a passive exoskeleton that provides antigravity support through a mechanical spring system (without actuators) within a three-dimensional workspace. The device allows movement along six degrees of freedom (DoF): three DoF at the shoulder to allow shoulder (flexion/extension, abduction/adduction, and internal/external rotation), one DoF at the elbow (flexion/extension), one DoF at the radioulnar joint (forearm pronation/supination) and one DoF at the wrist (wrist flexion/extension). A pressure-sensitive handgrip enables grip training. Participants’ movements are supported through the ArmeoSpring passive exoskeleton and translated via several different virtual reality (VR) tasks using the therapy software Armeocontrol (Version 2.2 or any newer stable version, Hocoma AG, Volketswil, Switzerland). The range of motion (ROM) needed to control the VR tasks is adjustable. Different VR tasks target different aspects of upper extremity function, including reaching movements in various directions, pronation/supination at the radioulnar joint and handgrip strength [12].

The device settings of the ArmeoSpring, including module length and antigravity weight support, are tailored to each participant. The virtual reality task difficulty is adjusted to each participants’ individual capabilities and aligned with the predefined goals. These settings are continuously reviewed and adapted throughout the intervention to maintain an appropriate level of challenge and promote task progression. There are no deviations from the original CE-marked instructions for use.

#### 2.4.2. Control Intervention OT

The unilateral conventional OT includes strengthening exercises (concentric and eccentric) with and without resistance, grasping exercises, fine motor training, peg games, and activity-based tasks that can be performed unilaterally (e.g., eating or brushing teeth). During these exercises, therapists provide only the support necessary to ensure correct execution. Therapy also focuses on weaker muscles by selectively recruiting the weaker muscles while preventing compensatory movements from stronger muscles.

### 2.5. Outcomes

#### 2.5.1. Primary Outcome

The primary outcome of this study is the GRASSP-QtG. The first version (V1) of GRASSP was introduced in 2009 by an international research group with the intention to enable sensitive and standardised assessment of upper extremity recovery in individuals with cSCI [19]. A revised version (V2) of GRASSP was later established with clarified instructions and a shorter format while preserving psychometric validity [20]. The GRASSP comprises four subtests, whereas the GRASSP-QtG subtest reflects upper extremity function based on quantitative measures of grasp performance. To assess the primary outcome, the V1 of the GRASSP-QtG is applied. It includes six prehension tasks which are performed in a strictly standardised way to provide a complete evaluation of grasp performance. Although GRASSP-QtG V2 was reduced to only four prehension tasks, all six tasks from GRASSP-QtG V1 are included to allow comparison with the predicted recovery profiles [16], which are based on GRASSP-QtG V1.

#### 2.5.2. Secondary Outcomes

The secondary outcomes include the three remaining subtests of GRASSP conducted according to GRASSP V2. These comprise the qualitative grasping, sensation and strength subtests [19,20]. By measuring all subtests of the GRASSP, a valid, reliable and responsive clinical upper extremity impairment measure in both acute and chronic cSCI will be conducted [20,21].

Additional secondary outcomes include independence in ADLs, measured by the Spinal Cord Independence Measure III–Self-report (SCIM III-SR), which is an effective measure for functional gains in rehabilitation [22,23], the Upper Extremity Motor Score (UEMS) of ISNCSCI, reflecting the degree of motor impairment of the upper extremities related to the SCI [18], as well as handgrip strength as a key indicator of hand functionality.

Furthermore, arm activity and motor control are assessed using the instrumented Action Research Arm Test (iARAT), which extends the standard ARAT [24] by integrating inertial measurement units (IMUs) as well as consumer-grade webcams (Logitech Brio, Logitech International S.A., Lausanne, Switzerland) operating at 60 Hz. This setup enables quantitative analysis of upper extremity kinematics [25]. In addition to the standardised ARAT tasks, predefined supplementary tasks are also recorded using IMUs and cameras.

#### 2.5.3. Further Outcomes

The therapy intensity of both interventions, RT and OT, is quantified. The acceleration and angular velocity measured by the IMUs allow for quantification of the therapy intensity. Given that RT is known for its highly intensive and repetitive exercises, therapy intensity can lead to a critical difference in improving upper extremity function compared to OT.

Motivational aspects are assessed using the Intrinsic Motivation Inventory (IMI), as RT incorporates gamified feedback designed to enhance engagement.

Therapy goals and their achievement by the end of the intervention block are documented. Goals are defined according to ICF and serve both to enhance motivation during the therapy sessions as well as to promote functional transfer to ADLs.

To provide a comprehensive view of the rehabilitation process, the frequency and content of clinical routine physical and occupational therapy as well as the interventional and control therapies are recorded.

The participants’ descriptive data include their demographics, SCI characteristics, medical history, medication and handedness to account for potential confounding variables.

All outcomes are described in detail in Table A1.

### 2.6. Data Management and Safety Provisions

#### 2.6.1. Data Management and Monitoring

For each enrolled participant, outcome measures are documented on paper-based case report forms (pCRFs). Any deviations from the protocol (e.g., missed or shortened sessions) are documented in the pCRF. The pCRFs are stored in a folder, which is kept in a lockable cupboard. The data on the pCRF are subsequently entered into electronic CRFs (eCRFs) in secuTrial. The secuTrial database ensures a permanent audit trail and complies with Good Clinical Practice (GCP) guidelines and regulatory requirements. The study database is managed by the study-independent data manager of the CTU. Prior to release into the productive environment, the randomisation, project set-up, and eCRFs are tested by the data manager and at least one study team member using a custom testing protocol. The study team has only predefined rights for data entries according to their role in the study. The data of Armeocontrol, IMUs and the videos are stored locally on the devices and then transferred to an encrypted hard-drive. A backup system is used and maintained by the study team. All hard drives are kept in a lockable cupboard.

All data will be archived for a minimum of 20 years. Access to folders and encrypted hard drives is limited to authorised study members only. Each participant is assigned a unique identification code. Documents allowing the identification of participants are stored separately on a secure server.

Besides the data management, the CTU is also responsible for the study-specific monitoring. The monitoring plan is based on the risk-based approach described in the ADAMON project [26]. All source data and documents are accessible to monitors, and questions will be answered during monitoring visits. A monitoring report will be provided after every monitoring visit.

#### 2.6.2. Safety Provisions

RT for upper extremity rehabilitation after cSCI has been shown to be safe and feasible [4]. The ArmeoSpring is a CE-marked device which has been used for several years all around the world. RT using the ArmeoSpring is routinely performed with individuals with cSCI, and no study has reported any occurrence of side effects or adverse health events related to its use. The user manual of ArmeoSpring indicates the following list of potential mild side effects: muscle pain, joint pain, skin irritation and lesions caused by the cuffs, as well as fatigue. Additionally, it includes recommended measures to prevent them (e.g., wearing long-sleeved clothing). During all training sessions, specifically trained therapy staff, investigators or members of the study team will monitor participants to minimise any potential harm. Overall, no serious device-related side effects or long-term harm to the participants are anticipated.

The outcomes assessed in the present investigation, such as the GRASSP, SCIM III-SR, the ISNCSCI-UEMS, iARAT, the hand grip dynamometry and the IMI, are routinely performed assessments in daily clinical practice which entail no excessive risks.

Participants can withdraw from the study at any time without providing a reason, and withdrawal of consent will not affect their subsequent medical assistance and treatment. They have to be excluded in case of adverse reactions (e.g., skin integrity issues at contact points with orthoses of the exoskeleton) that cause discontinuation of the therapy, lack of compliance (e.g., regular voluntary absence from therapy sessions) or surgery on the randomised upper extremity during the course of the study that affects the upper extremity function, therapy intensity or frequency (e.g., orthopaedic, hand or tetrahand surgery). All serious adverse events and device deficiencies are documented and reported to the Sponsor-Investigator and the responsible Ethics Committee throughout the study.

### 2.7. Statistical Analysis Plan

This exploratory randomised crossover trial is designed to estimate the pattern and magnitude of intervention effects on upper extremity function in individuals with cSCI. While primarily exploratory, the design allows for formal testing of superiority if a sufficiently strong intervention effect is observed.

In total, the study will randomise 40 participants to one of two intervention sequences. The crossover design enables paired comparison of intervention effects. Hence, a sample size of 34 evaluable participants will allow detection of a minimal clinically important difference (MCID) of three points in the primary outcome GRASSP-QtG with a power of 80% and a significance level of 5% (two-sided), as long as the corresponding within-group standard deviation does not exceed six points (corresponding to a medium effect size). Based on the number of patients with cSCI in previous years [11] and accounting for a 15% attrition rate, the study aims for a recruitment duration of two and a half years after first patient in to reach the calculated sample size of 40 participants.

Descriptive statistics will summarise the baseline characteristics of the study participants. Even though the SCI population is characterised by a predominance of males (~70%) [27,28], the neurological and functional recovery profiles have been found to be comparable between genders [29]. Therefore, no gender-specific stratification is planned. However, gender balance will be ensured within the study team for data collection, analysis, and authorship.

The primary and secondary outcomes will be analysed as changes within each period using linear mixed models. A two-sided significance level of α = 0.05 will be used for superiority testing. The model will include the baseline value measured at the start of the study, TREATMENT as a categorical variable with two levels (RT and OT), and PERIOD as a categorical variable with two levels (first and second) to assess the differences in changes in GRASSP-QtG between timepoints (i.e., t_1_–t_0_ and t_2_–t_1_). In addition, subject- and stratum-specific random intercepts will account for within-subject and within-stratum correlation across timepoints. To assess potential carryover effects, a second model will additionally include the TREATMENT × PERIOD interaction and SEQUENCE as a categorical variable with two levels (RT-OT and OT-RT). No interim analyses are planned for the main outcomes.

The primary analysis of this trial will follow the intention to treat principle, including all subjects in the statistical analysis who are randomised and provide a baseline (t_0_) assessment. Additionally, per-protocol analyses of all endpoints will be conducted as exploratory analyses to evaluate the effect of unilateral RT intervention under ideal adherence conditions (>75%, i.e., at least 14 sessions per intervention). In general, participants who discontinue the intervention (e.g., fulfilling exclusion criteria, early discharge or complications) will be encouraged to participate in an unscheduled assessment at least for t_1_ (or a timepoint before the individual end of study) and to ideally provide an assessment at least for the primary endpoint. To account for partially missing outcome data, linear mixed models assuming data are missing at random will be applied. In case of entirely missing outcome data, multiple imputation will be performed. The potential impact of missing values will be discussed in the final report.

Data analysis will be performed using contemporary versions of the statistical software packages R (Version 4.5.1, R Foundation for Statistical Computing, Vienna, Austria) and RStudio (Version 2025.05, RStudio, PBC, Boston, MA, USA), or any newer stable version available at the time of data analysis.

### 2.8. Dissemination Policy

The results of this study will be published in appropriate scientific journals and presented at relevant conferences. The CONSORT (extension to randomised crossover trials) guidelines as well as the flow diagram (Supplement S3) for publishing study results [30,31] will be followed. If sex- or gender-specific effects are observed, they will be published in the final study report. The Sponsor will enter and publish a summary of the results of the clinical investigation in a publicly recognised register immediately after submitting the final report. The investigator will provide each participant with the lay summary of the results of the clinical investigation at the end of the study.

## 3. Strengths and Limitations

Despite the limited evidence for RT in upper extremity rehabilitation following cSCI, it is frequently used in clinical practice. Therefore, this randomised crossover study was designed to systematically probe the effects of RT compared to conventional OT on upper extremity function in individuals with intermediate cSCI.

For developing this randomised crossover study design, the results and specific limitations of previous studies were considered. The ArmeoSpring device was selected for the experimental intervention due to its established advantages of widespread clinical use [11] and the significant functional improvements previously observed in a subgroup with residual hand function [12]. In contrast to earlier RCTs [7,8], the current study requires stricter inclusion criteria regarding the enrolment timepoint (16 < t_0_ < 81 days post-injury). With this, the majority of motor recovery can be targeted, which occurs within the first 6–9 months [10]. Whereas Bertels, et al. [32] concluded in their review that a minimum duration of 8 weeks will lead to improvements in function and activity level, Zariffa, et al. [12] showed that planned sessions of 1 h 3–5 times a week were not feasible. Additionally, considering that the first additional RT or OT intervention block already starts around 3 months post-injury, the current study comprises 3 × 30 min training sessions per week for 6 weeks per intervention block. These fewer and shorter therapy durations were determined according to reported therapy sessions in the literature varying between 30 and 60 min 3–5 times per week for 4–6 weeks [7,8,12] and are in line with existing clinical practices described by Kuchen, et al. [11].

Applying the interventions in a crossover study design has multiple advantages. First, every participant receives both the experimental and control interventions, eliminating ethical concerns about the selection of the comparator or unequal intervention allocation. Regardless of sequence (RT followed by OT or vice versa), participants engage in an intensive 12-week unilateral training program in addition to clinical routine therapy scheme, potentially enhancing functional recovery of the respective upper extremity. Additionally, participants decide together with their therapist which upper extremity to include in the study. This allows them to prioritise either the more severely impaired side to maximise recovery potential or the less impaired side to promote functional independence.

Moreover, the crossover design enables a within-participant comparison. This reduces interindividual variability and the total sample size required to detect meaningful effects. This design also permits the evaluation of sequence effects, providing information if a certain intervention sequence is more favourable compared to the other. Because only one upper extremity is randomised while both arms continue clinical routine therapy, the design allows comparison between the trained and untrained upper extremity. It yields information about the overall benefit of the additional intensive therapies compared to clinical routine therapies as well as the effectiveness of RT compared to OT. These exploratory tests can help to define new hypotheses for future research.

Despite its strength, the crossover design cannot eliminate the carry-over effects. The carry-over effects are taken into account by focusing the analysis on the improvements within each intervention block rather than the absolute results at each assessment timepoint. Consequently, improvements during the first intervention block could influence outcomes in the second. However, a specific wash-out period is not feasible and ethically inappropriate as the reacquired functions are unlikely to decline during primary rehabilitation.

Furthermore, time during primary rehabilitation is a strong determinant of motor recovery and may therefore influence observed improvements during the first compared to the second intervention period. By including PERIOD and the baseline measurement in the linear mixed model, we account for overall time-related recovery effects and thereby reduce confounding by the underlying recovery trajectory when estimating the treatment effect. However, potential carryover effects will be explicitly assessed and considered in the interpretation of the results.

Additionally, whereas only one arm will be randomised to one of the two sequences of intervention blocks, both arms continue with the clinical routine therapy. Although the clinical routine therapy could affect the study objectives, it would not be ethical to restrict the clinical routine therapy during primary rehabilitation after cSCI. However, the influence on the primary objective is reduced by the within-subject comparison due to the crossover design, provided that the concomitant therapy does not differ systematically between the blocks.

Finally, by having stratified randomisation in the crossover design, new hypotheses can be generated from exploratory subgroup analysis. With this, more information about possible individualisation of therapy schedules dependent on the patients’ predicted recovery profiles can be provided in the future.

## 4. Outlook

This randomised crossover study is designed to provide long-awaited evidence on the effects of RT on upper extremity function during primary rehabilitation after cSCI. We hypothesise that unilateral RT, using the ArmeoSpring, will result in greater improvements in upper extremity function compared to unilateral conventional OT, each for 3 × 30 min per week for 6 weeks additional to clinical routine therapy.

With its sophisticated design, the study generates data that should be interpreted from the perspective of previous studies and hypotheses and discussed in the broadest context possible. At the same time, the generated data provide an excellent data basis for the formulation of new hypotheses to be tested in further research. Furthermore, the results may provide arguments for negotiation with third-party payers for this expensive form of therapy.

In conclusion, future individuals with upper extremity impairment due to cSCI may profit from the generated knowledge. This study allows evidence-based recommendations regarding the effectiveness of additional RT and OT, the optimal intervention, and the timing of the interventions. These insights facilitate more effective and individualised therapy schedules for individuals with cSCI.

## Figures and Tables

**Table 1:** Eligibility criteria.

Inclusion Criteria	Exclusion Criteria
Informed consent signed by the subject Traumatic or non-traumatic cSCI Inclusion between 16 and 81 days post-injury Primary rehabilitation at the SPC NLI: C1-T1 AIS: A–D ≥18 years of age Impairment of upper extremity function (GRASSP-QtG < 25 at baseline t_0_) Ability to sit for 60 min and perform training with the ArmeoSpring Stratification parameters (ISNCSCI-FDP and GRASSP-Delto) available	Inability to follow the procedures (e.g., due to language problems or psychiatric disorders) Severe concomitant neurological disease (e.g., traumatic brain injury, polyneuropathy) Concomitant neurodegenerative or progressive diseases (e.g., multiple sclerosis, amyotrophic lateral sclerosis, cancer) Severe concurrent medical disease or any other issue that would confound the results in the opinion of the investigator Impairment due to peripheral nerve lesions (e.g., brachial plexus injury) Orthopaedic limitations of the upper extremity (e.g., joint luxation) Device specific contraindications: ○ Orthosis cannot be fitted to the relevant arm ○ Bone instability (non-consolidated fractures, severe osteoporosis) ○ Non-stable vital functions: Pulmonary or cardio-pulmonary contraindications ○ Contraindicated sitting position Participation in other interventional trials

cSCI = cervical spinal cord injury, SPC = Swiss Paraplegic Centre, NLI = neurological level of injury, C1 = first cervical spinal cord segment, T1 = first thoracic spinal cord segment, AIS = American Spinal Injury Association Impairment Scale, GRASSP-QtG = Quantitative subtest of Graded Redefined Assessment of Strength, Sensibility, and Prehension, ISNCSCI-FDP = Flexor Digitorum Profundus muscle of the International Standards for the Neurological Classification of Spinal Cord Injury, GRASSP-Delto = Musculus deltoideus pars acromialis of GRASSP.

## Data Availability

No new data were created or analysed in this study. Data sharing is not applicable to this article.

## Supplementary Materials

The following supporting information can be downloaded at: https://www.mdpi.com/article/10.3390/mps9020031/s1, Supplement S1: SPIRIT checklist; Supplement S2: Participant information and informed consent form; Supplement S3: CONSORT flow diagram.

## Abbreviations

The following abbreviations are used in this manuscript:

ADL	Activity of Daily Living
AIS	American Spinal Injury Association Impairment Scale
CRF	Case Report Form (pCRF paper CRF; eCRF electronic CRF)
cSCI	Cervical Spinal Cord Injury
CTU	Clinical Trial Unit
C1	First cervical spinal cord segment
Delto	Musculus deltoideus pars acromialis
DoF	Degrees of Freedom
EHI	Edinburgh Handedness Inventory
EKNZ	Ethikkommission Nordwest- und Zentralschweiz
EMSCI	European Multicentre Study about Spinal Cord Injury
FDP	Flexor Digitorum Profundus Muscle
GAS	Goal Attainment Scale
GRASSP	Graded Redefined Assessment of Strength, Sensibility, and Prehension
GRASSP-QtG	Quantitative Grasping Subtest of GRASSP
iARAT	Instrumented Action Research Arm Test
ICF	International Classification of Functioning, Disability and Health
IMI	Intrinsic Motivation Inventory
IMU	Inertial Measurement Unit
ISCI PT-OT BDS	International Spinal Cord Injury Physical Therapy-Occupational Therapy Basic Data Set
ISNCSCI	International Standards for Neurological Classification of SCI
MCID	Minimal Clinically Important Difference
MMT	Manual Muscle Testing
MRC	Medical Research Council
NLI	Neurological Level of Injury
OT	Occupational Therapy
RCT	Randomised Controlled Trial
ROM	Range of Motion
RT	Robot-assisted Therapy
SCI	Spinal Cord Injury
SCIM III-SR	Spinal Cord Independence Measure III–Self-report
SPC	Swiss Paraplegic Centre
T1	First thoracic spinal cord segment
UEMS	Upper Extremity Motor Score
VR	Virtual Reality

## Appendix A

**Table A1:** Detailed description of outcomes.

Outcome	Test	Test Description	Assessment Timepoints
Grasp performance	GRASSP-QtG, Version 1	Six prehension tasks each graded from 0 (the task cannot be conducted at all) to 5 (the task is conducted without difficulties using the expected grasping pattern and upper extremity function is unaffected) for each arm according to the grasp used and completeness of the task within 75 s. The scores of the six tasks will be added up, giving a maximum possible QtG subtest score of 30 points for each side.	Baseline (t_0_), intermediate (t_1_), post (t_2_) and follow-up (t_3_) assessment
Qualitative upper extremity function	Qualitative grasping, sensation and strength subtest of GRASSP, Version 2	The qualitative grasping subtest: three prehension ability tasks, each graded from 0 (the task cannot be conducted at all) to 4 (the task is conducted without difficulties and appropriate force) for each arm. The maximal qualitative grasping subtest score is 12 points for each side. The sensation subtest: three palmar sensations, each graded between 0 and 4. In total, up to 12 points for each side. The strength subtest: isometric MMT for 10 muscles in each arm, each graded according to the MRC scale from 0 (no muscle contraction) to 5 (normal activity to resist against maximal resistance), summing up to maximally 50 points per side.	Baseline (t_0_), intermediate (t_1_), post (t_2_) and follow-up (t_3_) assessment
Independence in ADL	SCIM III-SR	The first domain “Self-Care” consists of six items and the scores range from 0 to 20. The second domain “Respiration and sphincter management” includes four items; the third domain “ Mobility” contains nine items, both with scores from 0–40. Thus, the total score of SCIM III-SR ranges from 0 to 100.	Baseline (t_0_), intermediate (t_1_), post (t_2_) and follow-up (t_3_) assessment
Upper Extremity Motor Score	ISNCSCI-UEMS	For this, MMT of elbow flexion, wrist extension, elbow extension, 3rd distal interphalangeal flexion, and 5th finger abduction is conducted and scored. Each score is graded between 0 and 5 on the MRC scale. The maximum score for each extremity is 25. Thus, the total score of ISNCSCI-UEMS ranges from 0 to 50 for the upper extremities.	Baseline (t_0_), intermediate (t_1_), post (t_2_) and follow-up (t_3_) assessment
Handgrip Strength	Dynamometer	For the isometric hand and grip strength measurements, the participants apply maximum force (measured in kg) to the hand dynamometer (Baseline BIMS Digital-Dynamometer (Fabrication Enterprises Inc., White Plains, NY, USA)).	Baseline (t_0_), intermediate (t_1_), post (t_2_) and follow-up (t_3_) assessment
Arm control	iARAT	Four subscales (grasp, grip, pinch and gross movements), graded based on correct movement, grasp configuration and 5 s time limit. Each task is graded between 0 and 3 points, giving a maximum of 57 points. During the iARAT, IMUs (Xsens DOT, Movella Inc., Henderson, NV, USA) worn on the wrist, the upper arm, and the trunk. In addition, cameras will record the different tasks to improve their kinematic analysis.	Baseline (t_0_), intermediate (t_1_), post (t_2_) and follow-up (t_3_) assessment
Therapy intensity	Amount of arm movement	Measured by wearable IMUs worn on the wrist, upper arm and trunk	Three times within the first, second and third 2-week block of each 6-week intervention
Intrinsic Motivation	IMI	The full IMI includes 45 items distributed across seven subscales, of which the applicable items and subscales can be chosen. Each item is graded between 1 (not at all true) to 7 (very true), out of which the total score can be calculated by summarizing all points.	Once within the first, second and third 2-week block of each 6-week intervention
Descriptives	Diverse	Demographics; SCI characteristics, medical history, medication, handedness by EHI	Enrolment; Changes of medication during the study are documented.
Therapy goals	GAS	One goal is based on “body function”, one on “body structure” and the third goal on the “activities” domain according to ICF. For this, the patients can choose from a set of the most common goals as described by Kuchen, et al. [11]. The therapy goals are used to motivate patients during their therapy sessions as well as to better relate the results.	Therapy goal definition in each first therapy session, the therapy goal achievement in each last therapy session
Therapy content	Diverse	Clinical routine: Frequency and duration per week of each kind of therapy; and International Spinal Cord Injury Physical Therapy-Occupational Therapy Basic Data Set (ISCI PT-OT BDS). Additional OT: ISCI PT-OT BDS with adapted time durations. Additional RT: Armeocontrol, Software of ArmeoSpring	The clinical routine physical and occupational therapy is documented three times within the first, second and third 2-weeks block of each 6-week intervention, the add-on therapy content is documented in every therapy session.

GRASSP = Graded Redefined Assessment of Strength, Sensibility, and Prehension; GRASSP-QtG = Quantitative Grasping Subtest of GRASSP; ADL = Activity of Daily Living; SCIM III-SR = Spinal Cord Independence Measure III–Self-report; MMT = Manual Muscle Testing; MRC = Medical Research Council; ISNCSCI-UEMS = Upper Extremity Motor Score of International Standards for Neurological Classification of SCI; iARAT = Instrumented Action Research Arm Test; IMUs = Inertial Measurement Units; IMI = Intrinsic Motivation Inventory; SCI = Spinal Cord Injury; EHI = Edinburgh Handedness Inventory; GAS = Goal Attainment Scale; ICF = International Classification of Functioning, Disability and Health; ISCI PT-OT BDS = International Spinal Cord Injury Physical Therapy-Occupational Therapy Basic Data Set; OT = Occupational Therapy; RT = Robot-assisted Therapy.
