# Supplementary material for: Effectiveness of Robot-Assisted Versus Conventional Occupational Therapy on Changes in Upper Extremity Function After Cervical Spinal Cord Injury (Armeo X-over Trial): Study Protocol of a Randomised Crossover Trial
Source: Methods Protoc. 2026 Feb 26;9(2):31. doi: 10.3390/mps9020031 (PMC13010677; doi:10.3390/mps9020031)
Supplement: Supplementary file 1 [file mps-09-00031-s001.zip › 251121_S2_ICF_ArmeoX_StudyProtocol_MPs_V1.1.pdf]

Supplement 2: Participant information and informed consent form

Anfrage zur Teilnahme an medizinischer Forschung:

Schweizer Paraplegiker Forschung

Roboter-gestützte Therapie vs. konventionelle Ergotherapie für die oberen Extremitäten bei Personen mit einer Rückenmarksverletzung der Halswirbelsäule

Die Armeo X-over Studie

Sehr geehrte Dame, sehr geehrter Herr

Hiermit möchten wir Sie über die Armeo Crossover-Studie informieren und anfragen, ob Sie daran teilnehmen möchten.

In dieser Studie wollen wir herausfinden, welchen Effekt Roboter-gestützte Therapie (d.h. die Therapie durch Geräte mit passiver Gewichtsentlastung des Armes) verglichen zur konventionellen Ergotherapie, beides zusätzlich zum vorgegebenen Therapieplan, bei Personen mit einer Rückenmarksverletzung der Halswirbelsäule auf die Arm- und Handfunktion hat.

Ihre Teilnahme ist freiwillig. Alle Daten, die in diesem Projekt erhoben werden, unterliegen strengen Datenschutzvorschriften. Das Forschungsvorhaben wird durchgeführt von der Schweizer Paraplegiker-Forschung Nottwil. Bei Interesse informieren wir Sie gerne über die Ergebnisse aus dem Forschungsvorhaben.

In einem Gespräch erklären wir Ihnen die wichtigsten Punkte und beantworten Ihre Fragen. Die folgende Patienteninformation wiederholt zu Beginn das Wichtigste. Danach folgen die detaillierten Informationen zur gesamten Studie, sowie die Einwilligungserklärung.

Mit Ihrer Unterschrift bestätigen Sie, dass Sie die Patienteninformation gelesen und verstanden haben. Wenn Sie etwas nicht verstehen, fragen Sie bitte die Prüfperson. So nennen wir die Person, die für eine Studie verantwortlich ist und die Sie im Rahmen dieser Studie betreut.

Im Rahmen dieser Studie ist für Sie zuständig:

Chantal Wunderlin (Studienmitarbeiterin)
Schweizer Paraplegiker-Forschung
Guido A. Zäch-Strasse 4
6207 Nottwil
+41 41 939 66 15
chantal.wunderlin@paraplegie.ch

Dr. sc. ETH Mario Widmer (Prüfperson)
Schweizer Paraplegiker-Forschung
Guido A. Zäch-Strasse 4
6207 Nottwil
+41 41 939 51 97
mario.widmer@paraplegie.ch

Das Wichtigste in Kürze

1. Warum führen wir diese Studie durch?

Bei einer Rückenmarksverletzung der Halswirbelsäule wird konventionelle Ergotherapie, aber auch Roboter-gestützte Therapie (d.h. die Therapie durch Geräte mit passiver Gewichtsentlastung des Armes) als Standardtherapie angeboten. Damit soll die Funktion der Arme und Hände verbessert werden.

In dieser Studie wollen wir die Roboter-gestützte Therapie und die konventionelle Ergotherapie vergleichen und untersuchen, welche der beiden Therapieformen wirksamer bezüglich Verbesserung der Arm- und Handfunktion ist.

In **Kapitel 4** erfahren Sie mehr zum wissenschaftlichen Hintergrund der Studie.

2. Was müssen Sie tun, wenn Sie teilnehmen?

Wenn Sie sich entscheiden mitzumachen, werden Sie zufällig einer von 2 Gruppen zugeteilt. Zusätzlich zum normalen Therapiealltag führen Sie dann mit einem Ihrer Arme entweder zuerst 6 Wochen die Roboter-gestützte Therapie und dann 6 Wochen die Ergotherapie durch oder umgekehrt. Beide Therapieblöcke enthalten 3 x 30min Therapie pro Woche über je 6 Wochen zusätzlich zum normalen Therapiealltag. Somit enthält die Teilnahme an der Studie über 12 Wochen zusätzliche Therapie.

Um den Fortschritt der Arm- und Handfunktion zu dokumentieren, wird vor dem ersten Therapieblock, zwischen den beiden Therapieblöcken und nach Abschluss des zweiten Therapieblocks eine Untersuchung Ihrer Arm- und Handfunktion durchgeführt. Falls die Untersuchung nach Abschluss des zweiten Therapieblocks früher als 150 Tage nach Eintreten der Rückenmarksverletzung stattfindet, so wird 1 Monat danach noch eine weitere Untersuchung Ihrer Arm- und Handfunktion durchgeführt. Diese Untersuchungen dauern jeweils ungefähr 3 Stunden.

In **Kapitel 5** erfahren Sie mehr zum Ablauf und Vorgehen der Studie.

3. Welcher Nutzen und welches Risiko sind mit der Teilnahme verbunden?

Nutzen

Durch die Teilnahme an dieser Studie erhalten Sie die Möglichkeit, an einem strukturierten Trainingsprogramm für Ihre Arm- und Handfunktion teilzunehmen. Dieses regelmässige und intensive Training kann möglicherweise zu einer Verbesserung Ihrer Arm- und Handfunktion führen, wodurch Ihre Selbstständigkeit bei Alltagsaktivitäten gefördert werden kann. Allerdings

kann es auch sein, dass Sie keinen direkten Nutzen haben, wenn Sie an dieser Studie mitmachen. Mit Ihrer Teilnahme helfen Sie zudem künftigen Patientinnen und Patienten, so dass die Arm- und Handfunktion noch gezielter trainiert werden kann.

Risiko und Belastung

Roboter-gestützte Therapie wird als Standardtherapie in der Klinik verwendet und als sicher eingestuft. Mögliche Nebenwirkungen sind Muskelermüdung, Gelenkschmerzen, Druckstellen oder Hautverletzungen und Hautrötungen. Um diese Risiken zu minimieren, wird die Therapie immer durch eine*n geschulte*n Studienmitarbeiter*in begleitet, der*die auf die korrekte und sorgfältige Durchführung der Bewegungen achtet.

Sowohl die Roboter-gestützte Therapie als auch die Ergotherapie werden zusätzlich zum vorgegebenen Therapieplan durchgeführt, was zu einer erhöhten Belastung während insgesamt 12 Wochen führen kann.

In **Kapitel 6** finden Sie weitere Informationen zu Risiken und Belastungen.

Darum geht es im Detail: Informationen zur Studie

4. Der wissenschaftliche Hintergrund der Studie

Unser Forschungsvorhaben bezeichnen wir in dieser Informationsschrift als Studie. Wenn Sie an dieser Studie teilnehmen, sind Sie eine *Studienteilnehmerin* bzw. ein *Studienteilnehmer*.

4.1 Hintergrund: Warum führen wir diese Studie durch?

Nach einer Querschnittlähmung auf Höhe der Halswirbelsäule ist die motorische und auch sensorische Funktion beeinträchtigt. Dies führt zu Einschränkungen in den oberen Extremitäten, wodurch auch die Alltagsaktivitäten und die Selbstständigkeit limitiert sind. Um die Lebensqualität von Personen mit einer Rückenmarksverletzung bestmöglich zu verbessern, wird so früh wie möglich mit der Rehabilitation begonnen. Ziel der motorischen Therapie ist es, möglichst viele Wiederholungen auf einem an die eigenen Möglichkeiten angepassten Niveau durchzuführen, um die Funktion von Armen und Händen zu verbessern. Dafür ist insbesondere die Roboter-gestützte Therapie geeignet.

Zurzeit wird die Roboter-gestützte Therapie in der Rehabilitation nach einer Rückenmarksverletzung der Halswirbelsäule vereinzelt angewendet. Allerdings gibt es keine eindeutigen wissenschaftlichen Grundlagen, ob und in welchem Umfang Personen mit einer Rückenmarksverletzung der Halswirbelsäule von der Roboter-gestützten Therapie im Vergleich zur Ergotherapie ohne Gerät profitieren.

Wir wollen daher mit dieser Studie untersuchen, ob Roboter-gestützte Therapie einen grösseren Einfluss auf die Verbesserung der Arm- und Handfunktion hat als konventionelle Ergotherapie.

4.2 Aufbau der Studie: Wie gehen wir vor?

In unserer Studie werden wir entweder Ihren rechten oder linken Arm trainieren. Dabei dürfen Sie zusammen mit ihrem Therapieteam und der Prüfperson entscheiden, welche Körperseite im Rahmen dieser Studie trainiert werden soll.

Danach werden Sie zufällig in eine von 2 Gruppen eingeteilt. Man nennt dies Randomisierung. Dies ist wichtig, um verlässliche Ergebnisse zu erhalten.

Die eine Gruppe wird zuerst 6 Wochen lang die Roboter-gestützte Therapie durchführen und danach 6 Wochen lang die konventionelle Ergotherapie. Die zweite Gruppe beginnt hingegen mit der konventionellen Ergotherapie und wird als zweites die Roboter-gestützte Therapie durchführen. Durch die Randomisierung können wir herausfinden, welche Therapie besser wirkt, unabhängig davon, welche Therapieform als erstes durchgeführt wurde. Für beide Arme erhalten Sie im Übrigen weiterhin die im Rahmen der Erstrehabilitation üblichen Therapieeinheiten.

4.3 Regelungen zur wissenschaftlichen Forschung mit Menschen

Wir machen diese Studie so, wie es die Gesetze in der Schweiz vorschreiben (Humanforschungsgesetz, Datenschutzgesetze). Ausserdem beachten wir alle international anerkannten Richtlinien. Die zuständige Ethikkommission hat die Studie geprüft und bewilligt.

Unsere Studie ist eine nationale Studie. Das heisst, es gibt 40 Teilnehmende in der Schweiz.

Eine Beschreibung dieser Studie finden Sie auch auf der Internetseite des Bundesamtes für Gesundheit unter www.kofam.ch unter der SNCTP-Registriernummer SNCTP000006199 oder der BASEC-Nummer 2024-D0100.

5. Ablauf der Studie

5.1 Was müssen Sie tun, wenn Sie an der Studie teilnehmen?

Die Teilnahme an der Studie ist freiwillig und dauert ungefähr 4-5 Monate. Sie müssen sich an den Ablaufplan halten und auch an alle Vorgaben, die Ihre Prüfperson macht. Sie müssen Ihre Prüfperson informieren,

- wenn sich Ihr Gesundheitszustand ändert, z. B. wenn es Ihnen schlechter geht oder wenn Sie neue Beschwerden haben; dies gilt auch, wenn Sie die Studie vorzeitig abbrechen (→ Kapitel 5.3 und 5.4);
- wenn sich Ihre Medikation ändert.

5.2 Was passiert bei den Terminen?

Generell werden 3 Therapieeinheiten pro Woche geplant. Aufgrund des vorgegebenen Therapieplans und der unterschiedlichen Verfügbarkeiten der Therapeutinnen und Therapeuten, können auch zwischen 2 und 4 Therapieeinheiten pro Woche stattfinden. Insgesamt sind es aber nicht mehr als 18 Einheiten innerhalb von 6 Wochen. Ein Termin dauert ungefähr 30 Minuten. Die erste Therapieeinheit pro Therapieblock dauert zusätzliche 30 Minuten länger, so dass genügend Zeit vorhanden ist, um die jeweiligen Therapieziele und -inhalte zu besprechen. Zudem wird vor dem ersten Therapieblock, zwischen den beiden Blöcken und nach Abschluss des zweiten Therapieblocks eine Untersuchung Ihrer Arm- und Handfunktion durchgeführt. Falls die Untersuchung nach Abschluss des zweiten Therapieblocks früher als 150 Tage nach Eintreten der Rückenmarksverletzung stattfindet, so wird 1 Monat danach noch eine weitere Untersuchung Ihrer Arm- und Handfunktion durchgeführt. Diese Untersuchungen dauern jeweils ungefähr 3 Stunden. Die Abfolge der Termine ist im Ablaufplan weiter unten angegeben.

Bei allen Terminen machen wir Folgendes:

- Wir beantworten Ihre Fragen
- Wir stellen Ihnen Fragen zu Ihrem Gesundheitszustand

Bei einzelnen Terminen machen wir zudem:

- Wir legen Ihre eigenen Therapieziele fest
- Wir messen die Kraft und Funktion Ihrer Arme und Hände und der selbständigen Ausführung von Alltagsaktivitäten
- Wir messen Ihre Leistungen und die Intensität des Trainings mit speziellen Messgeräten (Sensoren) und Videoaufnahmen
- Wir befragen Sie zu Ihrer Motivation und zur Erreichung Ihrer Therapieziele

Ablaufplan

**Woche**	**0**	**1-6**	**7**	**8-13**	**14**	**(± 18)**
**Dauer (Std.)**	**3**	**9.5**	**3**	**9.5**	**3**	**(3)**
Untersuchung der Arm- und Handfunktion	+		+		+	(+)
Festlegen der Therapieziele		+		+		
Roboter-gestützte oder Ergotherapie		+		+		
Befragung zur Motivation		+		+		
Befragung zur Erreichung der Therapieziele			+		+	

Ablauf der Roboter-gestützten Therapie und Ergotherapie

Für die Roboter-gestützte Therapie wird der Arm in die Armorthese des ArmeoSpring (Hocoma AG, Volketswil, Schweiz) eingespannt (→ Abbildung 1). Der darin enthaltene passive Federmechanismus ermöglicht eine individuelle Gewichtsentlastung. Dadurch werden die Bewegungen in Form von spielerischen Übungen erleichtert, wodurch der Bewegungsumfang, Kraft und Koordination gesteigert werden können. Der ArmeoSpring ist geprüft, zertifiziert und wird regelmässig im klinischen Alltag angewendet.

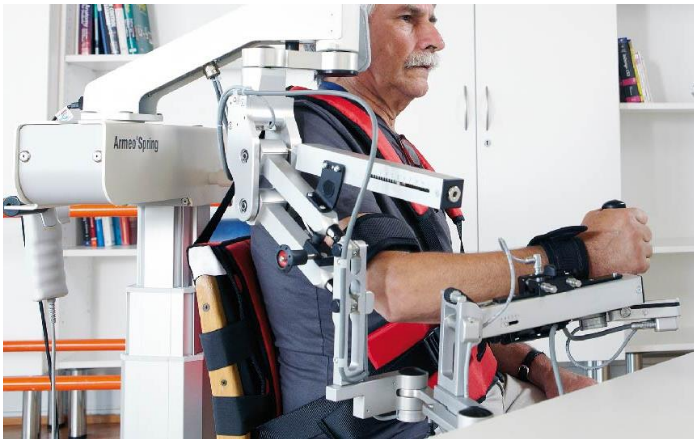

Abbildung 1: Die individuell anpassbare Armorthese des ArmeoSpring

In der konventionellen Ergotherapie werden hingegen Übungen für die Kraft und Feinmotorik sowie verschiedene Greiff- und Alltagsaktivitäten ohne zusätzliche Geräte oder Hilfsmittel durchgeführt.

5.3 Wann endet die Teilnahme an der Studie?

Für Sie dauert die Teilnahme 4-5 Monate und endet nach Abschluss des zweiten Therapieblocks, respektive 1 Monat danach, falls der Abschluss des zweiten Therapieblocks früher als 150 Tage nach Eintreten der Rückenmarksverletzung stattfindet.

Die Teilnahme ist freiwillig und Sie können Ihre Teilnahme jederzeit auch früher abbrechen (→ Kapitel 5.4). Sie müssen nicht begründen, warum Sie nicht mehr teilnehmen möchten. Zudem hat dies auch keinen Einfluss auf Ihre weitere medizinische Versorgung und Behandlung.

Wenn Sie selbst Ihre Teilnahme früher beenden möchten, sprechen Sie bitte mit Ihrer Prüfperson. In diesem Fall machen wir zu Ihrer Sicherheit abschliessend noch eine Untersuchung. Die bis dahin erhobenen Daten und Videoaufnahmen werden noch für die Studie ausgewertet. Ihre Studiendaten bleiben weiterhin verschlüsselt (→ Kapitel 9).

Es kann auch sein, dass wir Sie bitten müssen, die Studie frühzeitig zu beenden. Das ist zum Beispiel der Fall, wenn Sie aufgrund von Nebenwirkungen (z.B. Hautverletzungen bei den Kontaktpunkten zur Armorthese) die zusätzlichen Therapieeinheiten nicht mehr fortführen können. Ebenfalls müssen wir Sie bitten, die Studie frühzeitig zu beenden, falls Sie aus anderen Gründen nicht regelmässig an den Therapieeinheiten teilnehmen können. Dies gilt auch, falls Sie sich für einen chirurgischen Eingriff (z.B. Tetrahandchirurgie) am eingeschlossenen Arm während unserer Studienzeitdauer entscheiden, beziehungsweise unterziehen müssen.

5.4 Was passiert, wenn Sie nicht teilnehmen möchten?

Auch wenn Sie nicht an dieser Studie teilnehmen, behandeln und betreuen wir Sie medizinisch bestmöglich nach den aktuellen Standards.

6. Risiken, Belastungen und Nebenwirkungen

6.1 Welche Risiken und Belastungen können auftreten?

Es gibt Risiken und Belastungen bei der Teilnahme an dieser Studie, wie bei jeder medizinischen Behandlung. Sie finden in **Kapitel 6.2** eine Liste der häufigsten und schwersten Risiken. Viele Nebenwirkungen sind medizinisch behandelbar. Der ArmeoSpring ist ein geprüftes, zertifiziertes Gerät, welches regelmässig im klinischen Alltag verwendet wird. Die Therapie wird zudem immer durch eine*n geschulte*n Studienmitarbeiter*in begleitet, der*die auf die korrekte und sorgfältige Durchführung der Bewegungen achtet.

Zusätzlich gibt es Risiken bei den medizinischen Untersuchungen, die wir in dieser Studie machen. Manche Untersuchungen werden Sie bereits kennen. Sie finden in **Kapitel 6.3** eine Zusammenstellung dieser Risiken der Untersuchungen.

6.2 Die häufigsten und schwerwiegendsten Risiken durch die Interventionsmethode

Sie finden hier Informationen über die häufigsten und schwerwiegendsten Nebenwirkungen durch die Roboter-gestützte Therapie. Wir benutzen die folgenden Beschreibungen:

sehr häufig	Wir finden die Nebenwirkung bei mehr als 10 Personen von 100 (mehr als 10%).
häufig	Wir finden die Nebenwirkung bei 1 bis 10 Personen von 100 (1%-10%).
gelegent-lich	Wir finden die Nebenwirkung bei 1 bis 10 Personen von 1‘000 (0.1%-1%).
selten	Wir finden die Nebenwirkung bei 1 bis 10 Personen von 10‘000 (0.01%-0.1%).
sehr selten	Wir finden die Nebenwirkung bei weniger als 1 Person von 10‘000 (unter 0.01%).

Häufige, aber unbedenkliche Nebenwirkungen:

- Leichte Abschürfungen, Kratzer, Druckstellen, Blutergüsse, Hautirritationen oder Hautabschürfungen durch die Armhalterung des Roboters.

Gelegentliche, aber unbedenkliche Nebenwirkungen:

- Abschürfungen, Prellungen, Einklemmung des Schleimbeutels, kleinere Schnitte und Kratzer, Ausreißen eines Haarbüschels, Risse oder Spalten in den Fingernägeln und Schäden an der Kleidung durch Kollisionen mit dem Gerät vor, während und nach dem Training.
- Muskel- oder Gelenkschmerzen während und nach dem Training (spätestens nach ein paar Tagen), schmerzhafte Muskelanspannungen oder Muskelkrämpfe, Überdehnung bzw. Zerrung von Bändern, Sehnen und Muskeln, Schmerzen durch eventuelle Gelenkschäden bei wiederholter Durchführung sowie vorzeitige Ermüdung.

Selten und unbedenkliche Nebenwirkungen:

- Allergische Reaktionen, Hautreizungen sowie Übertragung von Infektionserregern zwischen Patienten durch die Berührung des Roboters.
- Kribbeln oder Muskelkrämpfe (infolge eines unerwünschten Leckstroms), leichte Verbrennungen oder eine vorübergehende Reizung der Atemwege (im unwahrscheinlichen Fall der Entwicklung von Rauch oder Feuer) durch elektronische Anzeigen (und damit Elektrizität) im Roboter.

Sehr selten, aber gefährliche Nebenwirkungen:

- Auskugeln der Schulter bzw. eines anderen Gelenks, Riss eines Bandes, Sehne oder Muskels sowie Prellungen, Blutergüsse und Knochenbrüche, auch im Bereich des Gesichts bzw. der Augen durch die beweglichen und frei hängenden Teile.
- Lokales Absterben von Gewebe an exponierten Hautstellen (Vernarbung) durch Verwendung von Kunststoffmaterialien.

6.3 Risiken und Belastungen durch Untersuchungen in der Studie

Die Untersuchungen im Rahmen dieser Studie beinhalten klinische Tests zur Messung von Kraft und Funktion der Arme und Hände, und zur selbständigen Ausführung von Alltagsaktivitäten. Diese Untersuchungen sind bewährte Verfahren und es bestehen nur geringfügige Risiken und Belastungen. Dazu gehören Muskelermüdung, Muskel- oder Gelenkschmerzen. Alle verwendeten Geräte sind zudem zertifiziert und für die Verwendung in der Schweiz zugelassen.

7. Finanzierung und Entschädigung

Die Studie wird von der Schweizer Paraplegiker-Stiftung und der Schweizer Paraplegiker-Forschung bezahlt und vom Schweizer Paraplegiker-Zentrum unterstützt. Die beteiligten Forschenden haben keinen unmittelbaren finanziellen Vorteil an der Durchführung dieser Studie.

Wenn Sie bei dieser Studie mitmachen, erhalten Sie jeweils CHF 50 für die Untersuchungen der Arm und Handfunktion, die an 3 bzw. 4 Zeitpunkten stattfinden. Die Teilnahme an den Therapieeinheiten wird nicht entschädigt.

Durch die Teilnahme an der Studie entstehen keine zusätzlichen Kosten für Sie oder für Ihre Krankenkasse.

8. Ergebnisse aus der Studie

Es gibt Ergebnisse, die Sie selbst betreffen. Diese Ergebnisse teilt Ihnen Ihre Prüfperson mit.

Es gibt auch Zufallsbefunde. Zufallsbefunde sind «Begleit-Ergebnisse», die nicht beabsichtigt sind. In dieser Studie erwarten wir keine Zufallsbefunde. Wir informieren Sie, falls trotzdem nicht zu erwartende Zufallsergebnisse auftreten sollten, die relevant sind für Ihre Gesundheit. Wenn Sie nicht informiert werden wollen, besprechen Sie das bitte mit Ihrer Prüfperson. Manche Zufallsbefunde werden immer mitgeteilt, zum Beispiel, wenn andere Personen gefährdet werden oder wenn es gesetzlich gemeldet werden muss.

Es gibt auch die Gesamtergebnisse der Studie, die aus den Daten von allen Teilnehmenden kommen. Dazu gehört zum Beispiel, dass wir mehr wissen über die Wirksamkeit von Roboter-gestützter Therapie bei Personen mit einer Rückenmarksverletzung der Halswirbelsäule (→

Kapitel 4.1). Diese Ergebnisse betreffen Sie und Ihre Gesundheit nicht direkt. Ihre Prüfperson gibt Ihnen am Ende der Studie aber gern eine Zusammenfassung der Gesamtergebnisse der Studie, wenn Sie das wünschen. Ausserdem werden die Ergebnisse nach Ende der Studie in Laien-verständlicher Sprache veröffentlicht.

9. Schutz von Daten und Proben

Wir schützen Ihre Daten (z.B. Angaben wie die Diagnose aus Ihrer Krankengeschichte oder die Resultate der Armfunktionstests). Zum Schutz von Daten gibt es in der Schweiz strenge gesetzliche Regelungen.

Das schweizerische Datenschutzgesetz gibt Ihnen das Recht auf Auskunft, Berichtigung und Erhalt Ihrer Daten, die im Rahmen der Studie erhoben, verarbeitet und weitergeleitet werden. Diese Rechte können in Ausnahmefällen wegen anderer gesetzlicher oder regulatorischer Anforderungen nicht immer garantiert werden. Wenn Sie Fragen dazu haben, wenden Sie sich bitte an Ihre Prüfperson.

9.1 Verschlüsselung von Daten

Bei jeder Studie entstehen Daten aus den Untersuchungen (z.B. Resultate von Armfunktionstests). Diese Daten werden dokumentiert. Das passiert in Papierform und elektronisch in grossen Tabellen, den sogenannten «Datenerhebungsbögen». Alle Daten werden verschlüsselt dokumentiert. «Verschlüsselt» heisst, dass persönliche Informationen, die Sie direkt identifizieren können, getrennt von den Untersuchungsergebnissen aufbewahrt werden. Dazu gibt es eine Liste (Schlüsselliste), die jede Person mit einem eindeutigen Code identifiziert. So stehen z.B. Ihr Name, Ihr Geburtsdatum oder Ihr Wohnort nicht direkt im Datenerhebungsbogen. Diese Schlüsselliste bleibt für die Dauer von 20 Jahren im Schweizer Paraplegiker-Zentrum Nottwil. Niemand sonst bekommt diese Schlüsselliste. Spezielle Ausnahmen sind in Kapitel 9.5 geregelt.

Die Videodaten werden für eine detaillierte Bewegungsanalyse automatisch ausgewertet. Die Teilnehmer*innen werden durch Unschärfe und Verpixeln des Bildes unkenntlich gemacht. Die so bearbeiteten Videodaten werden zusammen mit Ihren restlichen Untersuchungsdaten aufbewahrt und sind nur für Personen aus dem Studienteam zugänglich.

Die aufgenommenen Videodaten werden nur für Forschungszwecke verwendet, ausser es wurde explizit erlaubt, Teile des Videomaterials in der Öffentlichkeit (Präsentationen, Konferenzen, …) zu verwenden. Die Genehmigung zur öffentlichen Verwendung von verpixeltem Videomaterial können Sie am Ende der Einwilligungserklärung ausdrücklich geben oder verweigern.

Wenn wir Daten zum Zwecke dieser Studie weitergeben – zum Beispiel an andere Fachpersonen oder Organisationen, die weitere Untersuchungen machen – dann sind die Daten immer verschlüsselt und Ihre persönlichen Daten sind geschützt. Das gilt auch, wenn die Daten ins Ausland weitergegeben werden.

9.2 Sicherer Umgang mit den Daten während der Studie

Der Sponsor ist verantwortlich für den sicheren Umgang mit Ihren Daten aus dieser Studie. Er ist verantwortlich dafür, dass die geltenden Gesetze, z.B. die Datenschutzgesetze, eingehalten werden. Dies gilt auch, wenn verschlüsselte Daten für Untersuchungen in Länder verschickt werden, wo die Datenschutzgesetze weniger gut sind. So schützt der Sponsor dieser Studie Ihre Daten:

In dieser Studie werden Ihre Daten elektronisch erfasst und übermittelt. Die Daten sind auf einem Server in der Schweiz gespeichert. Trotzdem gibt es immer ein gewisses Restrisiko, dass fremde Personen auf Ihre persönlichen Daten zugreifen (z.B. Risiko von „Hacking“).

9.3 Sicherer Umgang mit Daten nach Ende der Studie

Der Sponsor bleibt auch nach Ende der Studie verantwortlich für den sicheren Umgang mit Ihren Daten. Das Gesetz schreibt vor, dass alle Studiendokumente (z.B. die Datenerhebungsbögen) und Videodaten für mindestens 20 Jahre aufbewahrt werden.

Nach Ende dieser langen Zeit bleiben Studiendaten verschlüsselt. Gesundheitsrelevante Daten Ihrer Krankengeschichte, auch von dieser Studie, sind und bleiben für Ihre Behandelnden immer zugänglich.

Nach Abschluss einer Studie werden die Ergebnisse meist in wissenschaftlichen Zeitschriften veröffentlicht. Dazu werden die Ergebnisse durch andere Fachpersonen begutachtet. Ihre verschlüsselten Daten müssen dabei an diese Fachpersonen weitergeleitet werden. Die Daten dürfen allerdings nicht für neue Forschungszwecke weiterverwendet werden. Dafür würde es Ihre separate Einwilligung brauchen (→ Kapitel 9.4).

9.4 Weiterverwendung und Weitergabe Ihrer Daten in anderen, zukünftigen Studien

Ihre Daten aus dieser Studie sind für die zukünftige Forschung sehr wichtig. Daten, welche für diesen Versuch verwendet wurden, können möglicherweise für andere Versuche weiterverwendet und/oder weitergegeben werden (auch ins Ausland).

Für die Weiterverwendung und/oder Weitergabe Ihrer Daten brauchen wir Ihre separate Einwilligung. Diese ist freiwillig. Bitte lesen Sie die zusätzliche Einwilligungserklärung am Schluss des Dokuments genau durch. Unterschreiben Sie bitte die Einwilligung, wenn Sie mit Ihren Daten weitere Forschung in der Zukunft unterstützen möchten. Auch wenn Sie nicht zustimmen, können Sie trotzdem an der Studie teilnehmen.

9.5 Einsichtsrechte bei Kontrollen

Die Durchführung dieser Studie kann überprüft werden. Die Überprüfung geschieht durch Behörden wie die zuständige Ethikkommission. Auch der Sponsor muss solche Überprüfungen machen, damit die Qualität dieser Studie und die Ergebnisse gesichert sind.

Dafür erhalten wenige, speziell dafür ausgebildete Personen Einblick in Ihre persönlichen Daten und in Ihre Krankengeschichte. Für diese Überprüfung sind die Daten also *nicht* verschlüsselt. Die Personen, die Ihre unverschlüsselten Daten sehen, unterliegen der Schweigepflicht.

Als Studienteilnehmer*in haben Sie jederzeit das Recht, Ihre Daten einzusehen.

10. Versicherungsschutz

Falls Sie durch die Studie einen Schaden erleiden sollten, haftet die Schweizer Paraplegiker-Forschung, die die Studie veranlasst hat und für die Durchführung verantwortlich ist. Die Voraussetzungen und das Vorgehen sind gesetzlich geregelt.

Bei Schäden, die auf ein zertifiziertes, gemäss der Gebrauchsanweisung angewendetes Medizinprodukt oder auch bei Anwendung einer üblichen Therapie aufgetreten wären, gelten dieselben Haftungsregelungen wie bei einer Behandlung ausserhalb einer Studie. In einem solchen Fall übernimmt die Haftpflichtversicherung der Schweizer Paraplegiker-Forschung die Kosten/Entschädigung.

Sollten Sie durch die Teilnahme an dieser Studie einen Schaden erleiden, so wenden Sie sich bitte an Ihre Prüfperson.

Einwilligungserklärung zur Teilnahme an einer Studie

Bitte lesen Sie dieses Formular sorgfältig durch. Bitte fragen Sie uns, wenn Sie etwas nicht verstehen oder wenn Sie noch etwas wissen möchten. Für die Teilnahme ist Ihre schriftliche Einwilligung notwendig.

**BASEC-Nummer**	2024-D0100
**Titel der Studie**	Robot-assisted vs. conventional occupational therapy of the upper limb in individuals with cervical spinal cord injury: The Armeo X-over Trial
**Laien-verständlicher Titel**	Roboter-gestützte Therapie vs. konventionelle Ergotherapie für die oberen Extremitäten bei Personen mit einer Rückenmarksverletzung der Halswirbelsäule: Die Armeo X-over Studie
**Verantwortliche Institution**	Schweizer Paraplegiker-Forschung<br>Guido A. Zäch-Strasse 4<br>6207 Nottwil
**Ort der Durchführung**	Schweizer Paraplegiker-Zentrum<br>Guido A. Zäch-Strasse 1<br>6207 Nottwil
**Verantwortliche Prüfperson am Studienort**	Dr. sc. ETH Mario Widmer
**Teilnehmerin / Teilnehmer:**<br>Name und Vorname in Druckbuchstaben:<br>Geburtsdatum:	

- Ich habe mündlich und schriftlich Informationen über die Studie bekommen, und zwar von der Prüfperson/dem Studienpersonal die/das unten unterschreibt.
- Die Prüfperson/das Studienpersonal hat mir den Zweck, den Ablauf und die Risiken der Studie erklärt.
- Ich nehme freiwillig an der Studie teil und ich hatte genügend Zeit, um diese Entscheidung zu treffen. Ich behalte die schriftliche Information und erhalte eine Kopie meiner schriftlichen Einwilligungserklärung.
- Ich kann jederzeit meine Teilnahme beenden. Ich muss nicht erklären, warum. Auch wenn ich die Teilnahme beende, bekomme ich weiter meine medizinische Behandlung. Die Daten und Videoaufnahmen, die bis dahin gesammelt wurden, werden im Rahmen der Studie noch ausgewertet.

- Wenn es besser für meine Gesundheit ist, kann mich die verantwortliche Prüfperson jederzeit von der Studie ausschliessen.
- Ich habe verstanden, dass meine Daten oder Videoaufnahmen nur in verschlüsselter bzw. verpixelter Form für diese Studie weitergegeben oder ins Ausland gesendet werden. Der Sponsor sorgt dafür, dass der Datenschutz nach Schweizer Standard eingehalten wird.
- Die zuständigen Fachpersonen der Ethikkommission dürfen meine unverschlüsselten Daten zur Kontrolle einsehen. Alle diese Personen unterstehen der Schweigepflicht.
- Bei Ergebnissen und/oder Zufallsbefunden, die direkt meine Gesundheit betreffen, werde ich informiert. Wenn ich das nicht wünsche, bespreche ich das mit meiner Prüfperson.
- Die Haftpflichtversicherung der Schweizer Paraplegiker-Forschung Nottwil kommt für allfällige Schäden auf.
- Ich erlaube, dass aufgezeichnetes Videomaterial in verpixelter Form zusätzlich zu internen Forschungszwecken auch öffentlich verwendet werden darf, zum Beispiel für Präsentation, Publikationen, Konferenzen oder ähnliches. ◯ Ja ◯ Nein

Ort, Datum	Name und Vorname Teilnehmerin / Teilnehmer<br>in Druckbuchstaben<br><br><br>Unterschrift Teilnehmerin / Teilnehmer

Bestätigung der Prüfperson / des Studienpersonals: Hiermit bestätige ich, dass ich dieser Teilnehmerin / diesem Teilnehmer Art, Bedeutung und Tragweite der Studie erläutert habe. Ich versichere, alle mit dieser Studie in Zusammenhang stehenden Verpflichtungen nach Schweizer Recht zu erfüllen. Sollte ich im Verlauf der Studie von Aspekten erfahren, welche die Bereitschaft der Teilnehmerin / des Teilnehmers zur Studienteilnahme beeinflussen könnten, werde ich sie / ihn umgehend darüber informieren.

Ort, Datum	Name und Vorname der Prüfperson / des Studienpersonals<br>in Druckbuchstaben<br><br><br>Unterschrift der Prüfperson / des Studienpersonals

Einwilligungserklärung für die Weiterverwendung und/oder Weitergabe von Daten in verschlüsselter Form

Diese Einwilligung betrifft Sie nicht im Sinne der persönlichen Teilnahme an einer Studie (→ Kapitel 9.4 der Patienteninformationen).

«Weiterverwendung» meint, dass Ihre Daten über die Zeit Ihrer Studienteilnahme hinaus aufbewahrt und in verschlüsselter Form für weitere Forschung verwendet werden können. Das kann z.B. heissen, dass eine Blutprobe und entsprechende Laborwerte von Ihnen zusammen mit einer grossen Zahl von anderen Werten statistisch ausgewertet werden oder neue Untersuchungen damit durchgeführt werden.

«Weitergabe» meint, dass Ihre Daten an andere Forschungspersonen oder Forschungsinstitutionen in verschlüsselter Form für weitere Forschungsprojekte weitergegeben werden dürfen. Diese anderen Forschungspersonen oder Forschungsinstitutionen können auch im Ausland angesiedelt sein. Es ist die Verantwortung des Sponsors, dass dieses Land über ein angemessenes Datenschutzniveau verfügt, welches mit der Schweiz vergleichbar ist.

**BASEC-Nummer:**	2024-D0100
**Titel der Studie**	Robot-assisted vs. conventional occupational therapy of the upper limb in individuals with cervical spinal cord injury: The Armeo X-over Trial
**Laien-verständlicher Titel**	Roboter-gestützte Therapie vs. konventionelle Ergotherapie für die oberen Extremitäten bei Personen mit einer Rückenmarksverletzung der Halswirbelsäule: Die Armeo X-over Studie
**Teilnehmerin / Teilnehmer:**<br>Name und Vorname in Druckbuchstaben:<br>Geburtsdatum:	

- Ich erlaube, dass meine verschlüsselten Daten und verpixelten Videodaten aus dieser Studie für die medizinische Forschung weiterverwendet und weitergegeben (auch ins Ausland) werden dürfen.
- Ich habe verstanden, dass die Daten verschlüsselt sind und der Schüssel sicher aufbewahrt wird.
- Die Daten können im In- und Ausland ausgewertet werden und in einer Datenbank hier oder im Ausland gespeichert werden. Forschungsinstitutionen im Ausland müssen dieselben Standards zum Datenschutz einhalten, wie sie in der Schweiz gelten.
- Ich entscheide mich freiwillig für die Weiterverwendung und/oder Weitergabe von Daten in verschlüsselter Form und kann diesen Entscheid zu jedem Zeitpunkt zurücknehmen. Ich

informiere lediglich die verantwortliche Prüfperson und muss diesen Entscheid nicht begründen.

- Wenn ich zurücktrete, bleiben die Daten verschlüsselt.
- Normalerweise werden alle Daten gesamthaft ausgewertet und die Ergebnisse zusammenfassend publiziert. Wenn sich zufällig ein Ergebnis zeigt, das für meine Gesundheit sehr wichtig ist, werde ich kontaktiert. Wenn ich das nicht wünsche, teile ich dies der verantwortlichen Prüfperson mit.

Ort, Datum	Name und Vorname Teilnehmerin / Teilnehmer<br>in Druckbuchstaben<br><br><br>Unterschrift Teilnehmerin / Teilnehmer

Bestätigung der Prüfperson / des Studienpersonals: Ich bestätige, dass ich der Teilnehmerin/dem Teilnehmer Art, Bedeutung und Tragweite der Weiterverwendung und/oder Weitergabe von gesundheitsbezogenen Daten erläutert habe.

Ort, Datum	Name und Vorname der Prüfperson / des Studienpersonals<br>in Druckbuchstaben<br><br><br>Unterschrift der Prüfperson / des Studienpersonals
