# Supplementary figures and images for: Effectiveness of Robot-Assisted Versus Conventional Occupational Therapy on Changes in Upper Extremity Function After Cervical Spinal Cord Injury (Armeo X-over Trial): Study Protocol of a Randomised Crossover Trial
Source: Methods Protoc. 2026 Feb 26;9(2):31. doi: 10.3390/mps9020031 (PMC13010677; doi:10.3390/mps9020031)

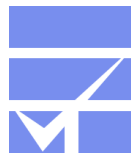

# CONSORT

## TRANSPARENT REPORTING of TRIALS

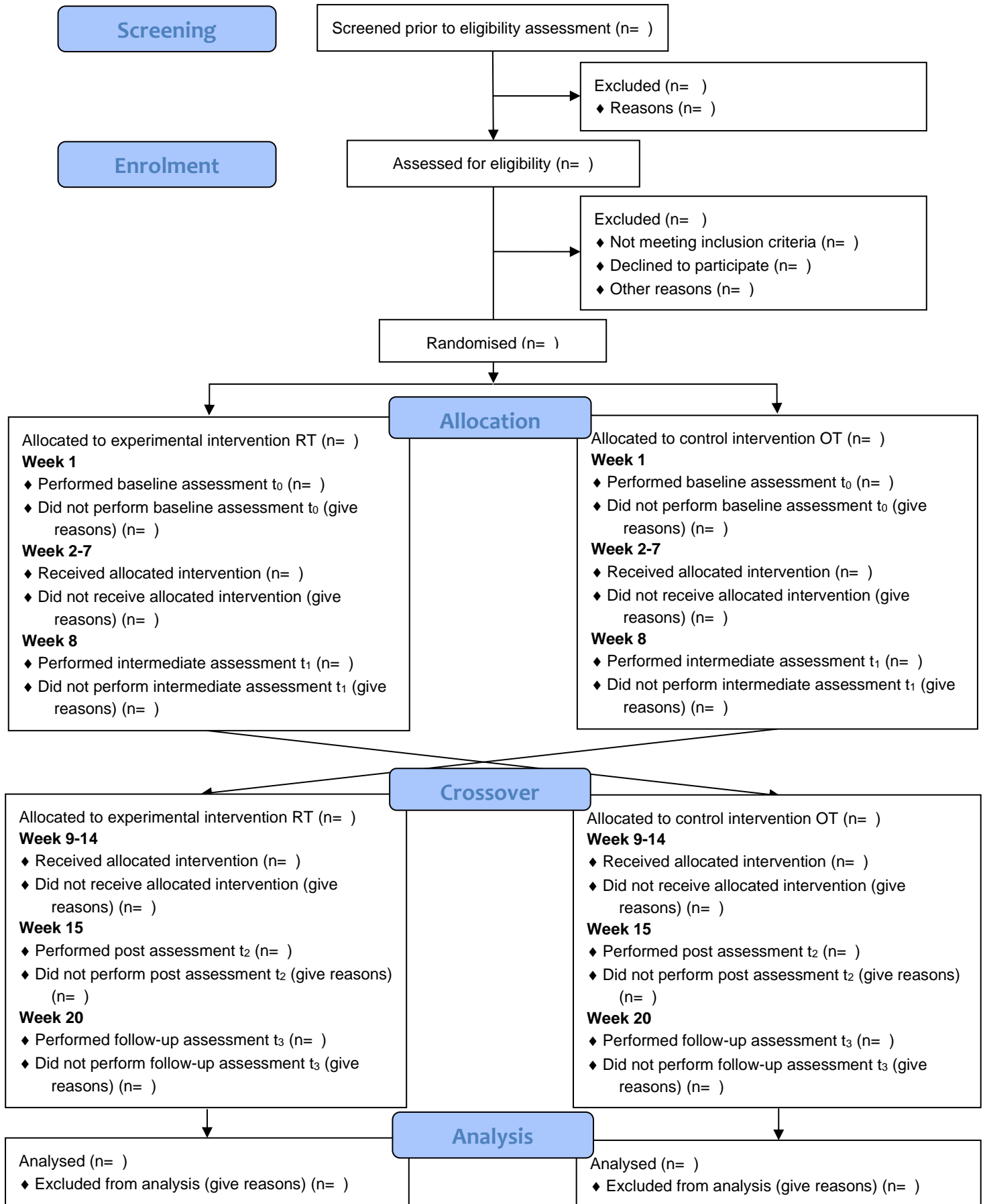

Supplement: Supplementary file 1 [file mps-09-00031-s001.zip › 251205_S3_CONSORT_ArmeoX_StudyProtocol_MPs_V1.0.pdf]
